# Integrated Multimodal Strategy to Reduce Healthcare-Associated Infections in a Trauma ICU: Impact of a Quality Improvement Project

**DOI:** 10.3390/jcm14165826

**Published:** 2025-08-18

**Authors:** Daiana Toma, Marius Păpurică, Alexandru Rogobete, Laura Andreea Ghenciu, Adelina Băloi, Claudiu Rafael Bârsac, Ovidiu Horea Bedreag, Carmen Alina Gizea, Ovidiu Alin Haţegan, Dorel Săndesc

**Affiliations:** 1 Anaesthesia and Intensive Care Research Center, Faculty of Medicine, “Victor Babes” University of Medicine and Pharmacy, 300041 Timisoara, Romania; daiana.toma@umft.ro (D.T.); papurica.marius@umft.ro (M.P.); adelina.baloi@umft.ro (A.B.); claudiu.barsac@umft.ro (C.R.B.); bedreag.ovidiu@umft.ro (O.H.B.); sandesc.dorel@umft.ro (D.S.); 2 Doctoral School, “Victor Babes” University of Medicine and Pharmacy, 300041 Timisoara, Romania; 3 Discipline of Pathophysiology, Department of Functional Sciences, “Victor Babes” University of Medicine and Pharmacy, Square Eftimie Murgu 2, 300041 Timisoara, Romania; 4 Center for Translational Research and Systems Medicine, “Victor Babes” University of Medicine and Pharmacy, Square Eftimie Murgu 2, 300041 Timisoara, Romania; 5 County Emergency Hospital ‘Pius Brinzeu’ Timisoara, 300723 Timișoara, Romania; carmalig@yahoo.com; 6 Discipline of Anatomy and Embriology, Medicine Faculty, Vasile Goldis Western University of Arad, Revolution Boulevard 94, 310025 Arad, Romania; hategan.ovidiu@uvvg.ro

**Keywords:** quality improvement project, nosocomial infections, intensive care unit, trauma patients, infection prevention, antimicrobial stewardship, mechanical ventilation

## Abstract

**Background**: Healthcare-associated infections (HAIs) remain a significant challenge in intensive care units (ICUs), especially in trauma settings where invasive interventions are frequent. This study aimed to assess the impact of a structured quality improvement project (QIP) on nosocomial infection rates and patient outcomes in a polytrauma ICU. **Methods**: We conducted a retrospective observational study at the “Pius Brînzeu” County Emergency Clinical Hospital, Timișoara. A total of 78 ICU trauma patients were included: 35 in the Pre-QIP group and 43 in the Post-QIP group. The QIP integrated evidence-based interventions, including hand hygiene reinforcement, individualized protective equipment, improved nurse staffing, and antimicrobial stewardship. Outcomes analyzed included nosocomial infection rate, ICU length of stay, antibiotic use, mechanical ventilation days, and mortality. Multivariable logistic, linear, and Poisson regression models were applied to control for confounding variables. **Results**: The Post-QIP group showed a significantly lower number of infections per patient (0.60 ± 0.95 vs. 1.41 ± 1.97, *p* = 0.03) and a trend toward lower mortality (0.19 vs. 0.34, *p* = 0.18). While ICU stay, antibiotic use, and ventilation days decreased post-QIP, these changes were not statistically significant. ISS and Charlson scores were consistent predictors of worse outcomes. **Conclusions**: Implementation of a targeted, multidisciplinary QIP was associated with improved infection control and patient outcomes. These results support the feasibility and value of structured infection prevention strategies in resource-constrained ICU settings.

## 1. Introduction

Healthcare-associated infections (HAIs), also known as nosocomial infections, represent a major global public health concern, significantly affecting morbidity, mortality, and healthcare costs. According to the most recent WHO global report, in low- and middle-income countries, 1 in 4 hospitalized patients acquires at least one HAI, and up to 1 in 10 dies as a result. Globally, 7 out of 100 hospitalized patients in acute care settings develop an HAI, with this proportion increasing to 15% in intensive care units [1]. According to ECDC data, the most frequently reported types of HAI in European hospitals are pneumonia (19%), urinary tract infections (18%), and catheter-related bloodstream infections (17%) [2].

Intensive care units are particularly vulnerable to HAIs due to the severity of patient conditions, underlying immunosuppression, and the frequent use of invasive devices such as central venous catheters, urinary catheters, and mechanical ventilation [3,4,5]. An international study published in 2020, including 15,202 patients from 88 countries, revealed that 54% of critical care patients had a suspected or confirmed infection, and 70% were receiving at least one antibiotic. The in-hospital mortality rate for patients with suspected or confirmed infection was 30%, emphasizing the major impact of infections on critically ill patients [6].

Reducing the incidence of HAIs is not only a quality improvement goal but also a professional and ethical duty for medical staff [7,8,9].

One of the most effective current strategies for preventing HAIs is the implementation of a quality improvement project (QIP), which involves applying a bundle of care—a set of evidence-based interventions implemented together, systematically and continuously [10,11,12,13]. According to the Institute for Healthcare Improvement, a care bundle is only considered effective if all components are delivered to each eligible patient every day [14]. International studies have shown that full adherence to such bundles is associated with significant reductions in infection rates and improvements in clinical outcomes [10,15].

In a Polytrauma Intensive Care Department of the “Pius Brînzeu” Emergency County Clinical Hospital in Timișoara, such a QIP has been implemented, including the following key measures: strict hand hygiene and adherence to general infection prevention protocols [16,17,18,19,20]; use of patient-specific disposable gowns by healthcare professionals and visitors [21,22,23]; optimization of nurse-to-patient ratios to reduce staff overload, a factor associated with increased infection risk [24,25,26,27,28]; and antibiotic stewardship in collaboration with infectious disease specialists, with the aim of treating only clinically confirmed infections and avoiding unnecessary treatment of positive cultures that may reflect colonization or contamination [29,30,31,32,33].

The implementation of multimodal infection prevention strategies, based on multidisciplinary team involvement and the application of evidence-based bundles, has proven to be effective in reducing HAIs [34,35,36]. A systematic review published in 2024 confirmed the effectiveness of such approaches. The authors emphasized that strategies combining staff education, regular audits with feedback, multidisciplinary team engagement, and standardized protocols significantly increase compliance and reduce infection rates [37]. Moreover, coordinated management involving intensivists, microbiologists, infectious disease specialists, and pharmacists has been shown to significantly improve outcomes in patients with Gram-negative bloodstream infections, both in terms of survival and microbiological clearance [38].

Despite the effectiveness of these interventions in international centers, underreporting of HAIs remains a major issue in Romania. A 2023 report by the WHO Country Office for Romania highlights the persistence of HAIs and the lack of consistent and accurate reporting despite existing legal obligations. These challenges are further compounded by systemic issues such as staffing shortages, inadequate infrastructure, lack of professional motivation, and fear of punitive measures—all of which hinder the effective implementation of infection prevention measures [39].

Nosocomial infections continue to be a major burden in intensive care settings. Recent data confirm that a substantial proportion of these infections is preventable. In the current context of rising antimicrobial resistance and increasing pressure on healthcare systems, structured and evidence-based approaches are crucial.

This study aims to evaluate the impact of a QIP implemented in a Polytrauma Intensive Care Department that integrates a coordinated bundle of care, including rigorous hygiene practices, individualized protective equipment, improved staff-to-patient ratios, and principles of antibiotic stewardship. The ultimate goal is to reduce the incidence of HAIs and enhance patient safety in intensive care through a model that can be adapted and replicated in similar clinical settings.

## 2. Materials and Methods

### 2.1. Study Aim

This study aimed to evaluate the impact of a structured infection prevention protocol on nosocomial infection rates and patient outcomes in an intensive care setting.

### 2.2. Study Design and Population

This retrospective, observational study was conducted in the intensive care unit of the Polytrauma Department at the “Pius Brînzeu” County Emergency Clinical Hospital in Timișoara. The study included patients admitted before and after the implementation of a QIP aimed at reducing nosocomial infections. The first group consisted of patients discharged between January and June 2020, before the intervention, while the second group included patients discharged between October 2021 and March 2022, after the intervention. Only trauma patients requiring intensive care were included, while non-trauma patients and those with ICU stays shorter than 48 h were excluded.

The two six-month periods were selected to ensure comparability in terms of ICU activity, seasonal variation in trauma admissions, and consistent availability of key resources, including staffing levels, equipment, and diagnostic support services. These intervals reflect standard clinical operation periods, excluding peaks of COVID-19 admissions that could bias results unrelated to the QIP intervention.

### 2.3. Data Collection and Variables

Clinical data were obtained from patient medical records and compiled into a structured database for analysis. The main variables included demographic characteristics, duration of hospitalization, severity scores, mechanical ventilation requirements, antibiotic use, and the incidence of nosocomial infections. Patients were divided into two groups based on the period of hospitalization, allowing for a comparative evaluation of infection rates and clinical outcomes before and after the intervention. Patient condition at ICU admission was assessed using the Injury Severity Score (ISS), which is a standardized measure of trauma severity at presentation and was recorded for all patients. In addition, comorbidity burden was evaluated using the Charlson Comorbidity Index (CCI), which was also recorded for all patients.

### 2.4. Quality Improvement Intervention

The study was conducted in a 6-bed intensive care unit at a Level I Regional Trauma Center (Clinical County Emergency Hospital “Pius Brînzeu”, Timișoara). Approximately 100 trauma patients are admitted annually, with 40–50% requiring ICU care. Our analysis included all trauma ICU admissions during two 6-month periods (pre- and post-QIP). IPC adherence was monitored through daily staff compliance checklists, routine audits of catheter and ventilator care, and antibiotic consumption logs. These measures allowed assessment of IPC implementation beyond just nosocomial infection rates. Detailed IPC interventions, including prevention strategies for ventilator-associated pneumonia, catheter-related bloodstream infections, and catheter-associated urinary tract infections, are summarized in Table 1.

The implemented quality improvement project focused on three key areas. First, infection prevention strategies included staff training on hand hygiene, proper use of personal protective equipment, and frequent environmental disinfection. Specific measures targeted infections related to mechanical ventilation, central venous catheters, and urinary catheters. Second, the nurse-to-patient ratio was optimized to improve patient care and adherence to infection control protocols. Third, an antibiotic stewardship program was introduced to regulate antibiotic use, ensuring appropriate treatment duration and reducing the risk of multidrug-resistant infections.

### 2.5. Statistical Analysis

Descriptive statistical methods were used to compare patient data between the pre-intervention group, pre-QIP (January–June 2020), and the post-intervention group, post-QIP (October 2021–March 2022). Continuous variables (age, ICU days, antibiotic use) were expressed as means ± standard deviation or medians with interquartile ranges, and group comparisons were conducted using Student’s *t*-test or Mann–Whitney U test depending on distribution normality. Categorical variables (sex, mortality) were analyzed using the Chi-squared test or Fisher’s exact test when appropriate. A *p*-value < 0.05 was considered statistically significant.

To identify independent predictors of in-hospital mortality, we performed a multivariable logistic regression analysis. Variables included in the final model were: QIP group status (Pre-QIP versus Post-QIP), age, sex, and Injury Severity Score (ISS). Additional variables, such as Charlson Comorbidity Index, ICU length of stay, number of infections, days on antibiotics, and mechanical ventilation days, were initially considered but excluded from the final model if they were likely to introduce post-treatment bias or were not significant after adjustment.

To assess the impact of the QIP intervention on ICU length of stay, mechanical ventilation duration, and antibiotic use, separate multivariable linear regression models were constructed. Each model included group status, age, sex, ISS, and Charlson Comorbidity Index as covariates.

For evaluating the effect of the QIP on nosocomial infection rates, a multivariable Poisson regression analysis was used, with the number of infections per patient as the outcome variable. Predictor variables included QIP group, age, sex, ISS, and Charlson Index.

All regression models were checked for multicollinearity, and interaction terms were explored. Results are reported as odds ratios (ORs), beta coefficients (βs), or incidence rate ratios (IRRs), along with 95% confidence intervals (CIs) and corresponding *p*-values.

Patients were categorized into two age groups: <65 years and ≥65 years. Injury severity was stratified using the Injury Severity Score (ISS), with ISS > 24 classified as severe injury. For each subgroup, we compared ICU days between the pre-QIP and post-QIP cohorts using both independent sample *t*-tests and Mann–Whitney U tests to account for potential non-normal distributions. All analyses were performed using IBM SPSS Statistics, version 27.0 (IBM Corp., Armonk, NY, USA).

## 3. Results

### 3.1. Patient Population Overview

A total of 78 patients were included in the study, with 35 admitted during the period preceding the QIP (pre-QIP group) and 43 admitted after its implementation (post-QIP group). Table 2 summarizes the baseline demographic and clinical characteristics of the two groups.

**Figure 1:**
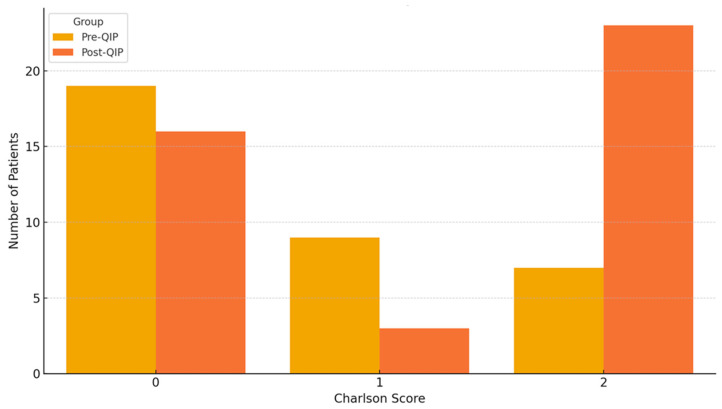
Distribution of Charlson Comorbidity Scores (pre-QIP/post-QIP).

Comorbidities were evaluated using the Charlson Comorbidity Index, which ranged from 0 to 2 in this patient population. The most commonly identified comorbidities were arterial hypertension, diabetes mellitus, chronic obstructive pulmonary disease, previous myocardial infarction, senile dementia, and malignancies. The distribution of Charlson scores across both groups is presented in Figure 1.

The total number of hospital-acquired infections (HAIs) decreased from 48 in the pre-QIP group to 26 in the post-QIP group. In the pre-QIP group, 15 out of 35 patients (42.85%) experienced at least one HAI. The most frequent infection types were bronchopneumonia (22 cases, 45.83%), bloodstream infections (14 cases, 29.16%), and urinary tract infections (12 cases, 25%). In the post-QIP group, 13 out of 43 patients (30.23%) developed HAIs. Bronchopneumonia remained the most common infection (16 cases, 61.53%), followed by bloodstream infections (6 cases, 23.07%) and urinary tract infections (4 cases, 15.38%). Overall, the implementation of the QIP led to a reduction in both the incidence and average number of HAIs per patient. Moreover, 10 patients in the pre-QIP group developed more than two HAIs compared to only 2 patients in the post-QIP group.

There were no significant differences in median age between the pre-QIP and post-QIP groups (47.5 ± 18.3 vs. 48.5 ± 19.4 years, *p* = 0.80). However, the sex distribution differed significantly, with a higher proportion of males in the pre-QIP group compared to the post-QIP group (85.7% vs. 60.5%, *p* = 0.02). Other baseline characteristics, such as ICU length of stay, ISS, Charlson Comorbidity Index, antibiotic use, and days on mechanical ventilation, did not differ significantly between groups (*p* > 0.05). The number of nosocomial infections per patient was significantly lower in the post-QIP group compared to the pre-QIP group (0.60 ± 0.95 vs. 1.41 ± 1.97, *p* = 0.03). Mortality also showed a numerical reduction in the post-QIP group (0.19 vs. 0.34), though this difference did not reach statistical significance (*p* = 0.18).

### 3.2. Multivariable Logistic Regression Analysis

A multivariable logistic regression analysis was performed to identify independent predictors of mortality among ICU patients. The model included the following clinical variables: patient group, age, sex, and ISS (Table 3). The aim was to determine whether the implementation of the QIP had a significant impact on reducing patient mortality, after adjusting for other potential confounding factors.

The results indicated that patients from the pre-QIP group had over 11 times higher odds of death compared to those in the post-QIP group (OR = 11.06, 95% CI: 0.98–124.70, *p* = 0.0519). Male patients had significantly increased odds of death compared to females (OR = 37.38, 95% CI: 1.27–1104.56, *p* = 0.0361).

Each one-year increase in age was associated with a 7% increase in the odds of death (OR = 1.07, 95% CI: 1.00–1.14, *p* = 0.0479), and higher ISS scores were also significantly associated with higher mortality (OR = 1.09 per point increase, 95% CI: 1.03–1.16, *p* = 0.0061).

Additional variables such as the Charlson Comorbidity Index, ICU length of stay, mechanical ventilation days, antibiotic use, and number of infections were initially considered for inclusion. However, they were excluded from the final model either due to their role as potential consequences of the outcome (introducing post-treatment bias) or because they did not demonstrate statistical significance when adjusted for more direct predictors like age and ISS score.

### 3.3. Impact of QIP on ICU Length Stay

To assess whether the QIP intervention affected ICU length of stay, we performed a multivariable linear regression analysis. The model included group status, age, sex, ISS, and Charlson Comorbidity Index (Table 4).

After adjusting for clinical and demographic factors, patients in the post-QIP group spent, on average, 4.37 fewer days in the ICU compared to those in the pre-QIP group (β = −4.37, 95% CI: −13.18 to 4.44), although this difference was not statistically significant (*p* = 0.32). Among other predictors, a higher Charlson Comorbidity Index was significantly associated with longer ICU stays (β = 8.60, 95% CI: 2.28 to 14.93, *p* = 0.008). ISS score showed a borderline association with longer ICU stay (*p* = 0.05), while age and sex were not significant predictors.

### 3.4. Impact of QIP on Nosocomial Infection Rates

We modeled the relationship between QIP implementation and nosocomial infection count using multivariable regression (Table 5). The number of infections per patient was used as the outcome variable, and the model included group status, age, sex, ISS, and Charlson Comorbidity Index as predictors. Patients in the pre-QIP group had a 22% higher rate of nosocomial infections compared to those in the post-QIP group (IRR = 1.22, 95% CI: 0.69–2.17), although this difference was not statistically significant (*p* = 0.49). However, male sex was significantly associated with a higher infection rate, with male patients exhibiting nearly four times the incidence of nosocomial infections compared to females (IRR = 3.94, 95% CI: 1.39–11.19, *p* = 0.009). Both greater injury severity (IRR = 1.03 per ISS point, 95% CI: 1.01–1.05, *p* = 0.005) and higher Charlson Comorbidity Index (IRR = 1.71, 95% CI: 1.15–2.55, *p* = 0.008) were also significantly associated with increased infection risk. Age was not significantly associated with infection count.

### 3.5. Effect of QIP on Antibiotic Use

We examined whether the implementation of the QIP was associated with changes in ICU antibiotic use using multivariable linear regression (Table 6). The number of days of antibiotic therapy per patient was used as the outcome, and group status, age, sex, ISS, and Charlson Comorbidity Index were included as predictors.

After adjusting for confounders, there was no statistically significant difference in antibiotic use between patients treated before and after the QIP intervention (β = −0.79, 95% CI: −7.24 to 5.65, *p* = 0.80). Similarly, age and sex were not significantly associated with antibiotic consumption. However, higher injury severity was significantly associated with increased antibiotic use: each point increase in ISS was linked to an average of 0.29 additional days of antibiotics (95% CI: 0.05 to 0.52, *p* = 0.01). Importantly, the Charlson Comorbidity Index showed a borderline significant association with longer antibiotic duration (β = 4.57, 95% CI: −0.05 to 9.20, *p* = 0.05).

### 3.6. Effects of QIP in Mechanical Ventilation Days

A multivariable linear regression model was applied to assess the effect of the QIP on the duration of mechanical ventilation (Table 7). The number of days of mechanical ventilation per patient was used as the dependent variable, and group status, age, sex, ISS, and Charlson Comorbidity Index were included as independent variables. Patients from the post-QIP group required, on average, 3.34 fewer days of mechanical ventilation compared to those in the pre-QIP group (β = −3.34, 95% CI: −11.51 to 4.84), though this difference was not statistically significant (*p* = 0.41). Similarly, neither age nor sex was significantly associated with ventilation duration. In contrast, both higher injury severity and greater comorbidity burden were significant predictors of prolonged mechanical ventilation. Each point increase in the ISS was associated with 0.32 additional ventilation days (95% CI: 0.02 to 0.61, *p* = 0.03), while each point increase in the Charlson Comorbidity Index was associated with an average 0.81-day increase in ventilation duration (95% CI: 0.08 to 1.55, *p* = 0.03).

### 3.7. Subgroup Analysis

A subgroup analysis was performed to assess differences in ICU length of stay between the pre-QIP and post-QIP cohorts, stratified by age group and injury severity.

Among patients aged <65 years, the mean ICU stay was slightly higher in the pre-QIP group compared to the post-QIP group; however, this difference was not statistically significant (*t*-test *p* = 0.70; Mann–Whitney U test *p* = 0.78). Similarly, in patients classified with severe injury severity (ISS > 24), the average ICU stay was longer in the pre-QIP group compared to post-QIP, but again, no statistically significant difference was observed (*t*-test *p* = 0.68; Mann–Whitney U test *p* = 0.68).

## 4. Discussion

HAIs remain a major cause of morbidity and mortality among critically ill patients, especially in trauma ICUs. Multiple studies have documented the disproportionately high incidence of healthcare-associated infections in intensive care environments, where the frequent use of invasive devices and the rising prevalence of antimicrobial resistance pose serious challenges to patient safety and treatment efficacy [12,40]. In our study, the introduction of a structured quality improvement initiative in our ICU was associated with a notable decline in the rate of HAIs, reflecting similar patterns of reduction observed in large-scale international programs focused on infection prevention and control [6,41].

Multinational data confirm that ICU patients are particularly vulnerable, with up to 54% presenting with suspected or confirmed infections and mortality rates exceeding 30% among these patients [1]. Considering the structural limitations often encountered in Eastern European healthcare systems—including workforce shortages, insufficient reporting mechanisms, and infrastructural deficiencies—our results highlight that structured, low-cost interventions based on standardized protocols can still be effectively implemented, even in settings with restricted resources. We aimed to integrate internationally validated best practices into a single, coordinated intervention tailored to the local clinical context.

Inspired by the care bundle framework proposed by Resar et al. [13], our team implemented a structured QIP within the Polytrauma Intensive Care Department of the “Pius Brînzeu” Emergency County Clinical Hospital in Timișoara. Multiple studies, including a systematic review and large institutional implementation project, have demonstrated that perioperative and device-specific bundles can significantly reduce the incidence of surgical site infections, ventilator-associated pneumonias, and catheter-related bloodstream infections [10,42]. Moreover, Furuya et al. demonstrated that compliance above 95% with central line bundles correlated with the greatest reductions in bloodstream infections [43].

The consistent implementation of care bundles has emerged as a cornerstone strategy in reducing healthcare-associated infections in intensive care units. Their effectiveness lies not only in the scientific validity of each individual component but also in the structured and simultaneous application of all measures included in the bundle. This integrative approach ensures that critical preventive actions are not applied in isolation, but rather as part of a cohesive and reproducible protocol that reinforces adherence and minimizes variation in clinical practice. As emphasized by Timsit et al. [44], standardization and high compliance remain essential to maximizing the preventive impact of such interventions. Ultimately, care bundles serve as practical tools for translating evidence-based guidelines into daily ICU workflows, fostering a culture of accountability and safety that is indispensable for sustained infection control.

The sustained effectiveness of bundle-based interventions is closely linked to their integration within a collaborative, multidisciplinary framework. As highlighted by Rinaldi et al. [38], the involvement of a dedicated team—intensivists, infectious disease specialists, clinical pharmacologists, and microbiologists—not only optimized antimicrobial therapy but also enhanced adherence to infection prevention protocols. Their findings emphasize that institutional engagement and coordinated team-based management are essential elements for translating bundle compliance into meaningful clinical outcomes, particularly in high-risk ICU populations.

This perspective is further reinforced by the findings of Potugari et al., who demonstrated that a sustained reduction in central line-associated bloodstream infections was achievable through a dual approach that combined leadership support with frontline staff engagement in a multidisciplinary framework [45]. By fostering collaboration between infection prevention specialists, nursing staff, and ICU clinicians, the intervention effectively bridged policy and practice, ensuring that protocol adherence translated into consistent bedside implementation.

In our QIP, although a formal multidisciplinary team was not institutionalized, stewardship principles were followed, and infectious disease specialists provided consultative support. Such a type of informal yet protocol-driven collaboration functioned similarly to formal multidisciplinary rounds, demonstrating that while the organizational structure may differ, the presence of coordinated, team-based implementation remains essential for achieving consistent infection control outcomes.

A critical structural factor in the success of infection control is the nurse-to-patient ratio. When staffing levels are insufficient, essential components of care bundles may be omitted, opportunities for hand hygiene can be overlooked, and timely clinical interventions may be delayed. The association between inadequate nurse staffing, increased missed care, and adverse patient outcomes has been consistently documented in the literature. A large multicenter European study found that higher patient-to-nurse ratios were significantly associated with greater frequency of missed nursing care, which in turn was linked to increased postoperative mortality [28,46]. Notably, Romania was among the countries that participated in this study, contributing data under the same standardized protocol. This inclusion reinforces the relevance of the findings to the Romanian context, where staffing shortages remain a persistent challenge in intensive care settings.

Beyond staffing numbers alone, the quality of the nursing work environment plays a pivotal role in infection prevention and overall patient safety. While quantitative metrics such as nurse-to-patient ratios provide a structural foundation, they do not fully capture the contextual factors that influence clinical performance. Lake et al. confirmed that improved nursing environments are predictive of lower infection rates and better patient safety outcomes [26].

In our setting, optimizing nurse-to-patient ratios was pivotal in supporting care bundle adherence. Tencic and Roche’s study reinforced that adherence to infection control practices increased as workloads decreased [25]. This human factor cannot be overlooked in any QIP aimed at infection reduction. In Romania, ICU staffing is regulated by Order no. 1500/2009, which sets minimum nurse staffing norms by ICU category and bed type. For intensive care patients, the standard translates to roughly one nurse per two to four beds, while for intermediate care, it can be one per four to six beds, depending on unit type and case mix [47]. Internationally, standards are similar: UK guidance (British Association of Critical Care Nurses) recommends 1:1 for Level-3 patients and 1:2 for Level-2 [48], and the American Association of Critical-Care Nurses (AACN) advises that most critically ill patients need at least 1:2 [49]. Maintaining adequate nurse staffing is strongly linked to better outcomes in critical care, fewer errors, faster responses to changes in patient condition, and improved overall clinical performance. Before the intervention, our ICU operated at approximately one nurse to four patients, which is considered in the lower range of optimal standards for high-acuity trauma care. Following staffing reinforcement, the ratio improved to 1:2, matching the upper range of recommended standards.

Another essential component of our intervention was the implementation of antimicrobial stewardship measures, particularly focused on optimizing the timing and appropriateness of empiric therapy in critically ill patients. In the intensive care setting, delays or inaccuracies in the initial selection of antimicrobial agents can have profound consequences. This is particularly critical in intensive care settings, where delayed or inappropriate empiric antimicrobial therapy has been strongly associated with increased mortality, and overuse of broad-spectrum antibiotics drives resistance [50]. Stewardship interventions are known to reduce antibiotic consumption, minimize resistance, and decrease costs without compromising patient outcomes [51].

Lindsay et al. [33] emphasize that structured antimicrobial oversight, particularly through prospective audit and feedback, can be safely implemented in intensive care settings and plays a critical role in optimizing antibiotic use. While their systematic review did not demonstrate a significant impact on ICU mortality, the findings suggest that integrating stewardship activities into daily clinical routines enhances the effectiveness of these interventions and supports more judicious antimicrobial prescribing.

In our cohort, antibiotic stewardship efforts sought to address precisely this vulnerability by promoting early, appropriate antimicrobial use while avoiding unnecessary exposure to broad-spectrum agents, a balance that is critical to both patient outcomes and resistance prevention. Within the context of our intervention, the QIP contributed to shorter antibiotic durations and improved targeting, although some findings did not reach statistical significance due to sample size limitations.

## 5. Sustainability, Limitations, and the Romanian Context

Ensuring the sustainability of infection prevention interventions requires ongoing engagement, education, and institutional support, elements that are often difficult to maintain in resource-limited settings. Moe Bell et al. [52] demonstrated that multidisciplinary staff education played a central role in reducing catheter-associated urinary tract infections, emphasizing the importance of continuous training and cross-functional collaboration in achieving long-term outcomes. Similarly, Vicentini et al. [34] highlighted the effectiveness of implementing multimodal strategies, combining surveillance, feedback, and environmental interventions, in reducing HAI prevalence across diverse clinical settings. However, applying these strategies in the Romanian context is not without challenges. Systemic issues such as underreporting, variability in local infrastructure, and limited human resources continue to hinder consistent implementation and monitoring. Despite these limitations, the present findings suggest that structured, evidence-based approaches remain feasible and potentially impactful even in constrained healthcare environments, provided that efforts are tailored to local realities and supported by ongoing institutional commitment.

Limitations of our study include its retrospective nature, single-center design, and the possibility of residual confounding due to variables not captured in our dataset, such as detailed comorbidity profiles beyond the Charlson score or specific infection management nuances. The use of two predefined six-month intervals, although chosen to align ICU activity, account for seasonal patterns in trauma admissions, and match staffing and resource conditions, may still introduce period selection bias. This is because short, fixed time frames may not fully capture the variability in patient case-mix, epidemiologic trends, or operational pressures that occur over longer observation periods. In addition, the relatively short six-month duration per period restricts the ability to observe sustained effects of the intervention or identify delayed outcomes. The sample size, while adequate for primary analyses, was modest and may have limited statistical power for detecting smaller effect sizes. Despite these limitations, the internal consistency across multiple outcome measures, such as a significant reduction in nosocomial infection rates, decreased antibiotic consumption, and a trend toward reduced ICU mortality, provides supportive evidence of the beneficial impact of the QIP.

Future directions should include scaling the intervention across ICUs in Romania, adding real-time electronic surveillance, and institutionalizing multidisciplinary structures to support sustainability.

## 6. Conclusions

The implementation of a structured QIP in the polytrauma intensive care setting led to a significant reduction in nosocomial infections and a trend toward improved clinical outcomes, including lower mortality, reduced mechanical ventilation days, and shorter antibiotic use. Although not all differences reached statistical significance, the overall findings support the effectiveness of coordinated, evidence-based interventions. This study highlights the feasibility and value of multidisciplinary, bundle-based approaches to infection control and provides a replicable model for similar ICUs facing comparable structural and organizational challenges.

## Figures and Tables

**Table 1:** Summary of quality improvement measures implemented in ICU.

Category	Specific Measures Implemented
Standard IPC Strategies	Hand hygiene per WHO’s 5 moments (training and monitoring)
	Mandatory PPE use: gloves, gown, cap for all patient contact
	Surface disinfection: 3× weekly (surfaces), 2–3× daily (floors)
	Use of sterile gloves for invasive/aseptic procedures
Targeted IPC Strategies	Ventilator-Associated Pneumonia (VAP):
	Daily oral care with chlorhexidine
	Daily sedation break, cuff pressure monitoring (20–30 cm H_2_O)
	Closed suction systems, subglottic suction ETT, passive humidification Sterile suctioning via orotracheal tube using sterile gloves and sterile suction catheter
	Central Line-Associated BSI (CLABSI):
	Maximal sterility during insertion, antimicrobial-impregnated catheters
	Dressing change every 7 days, port disinfection, daily IV set change
	Catheter-Associated UTI (CAUTI):
	Aseptic insertion, closed drainage system
	Catheter secured above thigh, bag below bladder, replaced every 14 days or sooner if needed
Staffing Optimization	Nurse-to-patient ratio improved from 1:4 (pre-QIP) to 1:2 (post-QIP)
Antibiotic Stewardship	Local antibiogram-guided de-escalation protocols
	Avoiding treatment solely based on positive cultures
	Regular screening for MDR colonization
	Monitoring and reduction of total antibiotic consumption

**Table 2:** Baseline characteristics and clinical outcomes of ICU patients before and after the QIP implementation.

Parameter	Pre-QIP Group (n = 35)	Post-QIP Group (n = 43)	*p*-Value
Age (years)	47.54 ± 18.29	48.49 ± 19.35	0.8
Male/Female (%)	85.71/14.29	60.47/39.53	0.02
ICU (days)	16.51 ± 22.12	15.35 ± 15.00	0.79
ISS Score	34.45 ± 14.80	31.29 ± 12.80	0.39
Charlson Comorbidity Index	0.76 ± 0.89	1.14 ± 0.91	0.07
Antibiotic Use	11.99 ± 17.89	9.00 ± 958	0.38
Mechanical Ventilation (days)	14.03 ± 21.59	12.35 ± 12.71	0.68
Nosocomial infection (number)	1.41 ± 1.97	0.60 ± 0.95	0.03
Mortality	0.34	0.19	0.18

Abbreviations: QIP—quality improvement project, ICU—intensive care unit, ISS—Injury Severity Score.

**Table 3:** Multivariable logistic regression analysis of factors associated with ICU mortality.

Variable	OR	95% CI	*p*-Value
Pre-QIP group (vs. post-QIP)	11.06	0.98–124.70	0.05
Male (vs. Female)	37.38	1.27–1104.56	0.03
Age (per year)	1.07	1.00–1.14	0.04
ISS score	1.09	1.03–1.16	0.006

Abbreviations: CI—confidence interval; OR—odds ratio.

**Table 4:** Multivariable linear regression analysis of factors associated with ICU length of stay.

Variable	Coefficient	95% CI	*p*-Value
Pre-QIP group (vs. post-QIP)	−4.37	−13.18 to 4.44	0.32
Male (vs. female)	3.38	−6.64 to 13.39	0.5
Age (per year)	−0.25	−0.56 to 0.06	0.10
ISS score	0.31	−0.01 to 0.63	0.05
Charlson Comorbidity Index	8.60	2.28 to 14.93	0.008

**Table 5:** Multivariable regression analysis of predictors of nosocomial infection rates.

Variable	IRR	95% CI	*p*-Value
Pre-QIP group (vs. post-QIP)	1.22	0.69–2.17	0.49
Male (vs. female)	3.94	1.39–11.19	0.009
Age (per year)	0.99	0.97–1.01	0.49
ISS score	1.03	1.01–1.05	0.005
Charlson Comorbidity Index	1.71	1.14–2.55	0.008

Abbreviations: IRR—incidence rate ratio.

**Table 6:** Multivariable linear regression analysis of factors associated with antibiotic use in the ICU.

Variable	Coefficient	95% CI	*p*-Value
Pre-QIP group (vs. post-QIP)	−0.79	−7.24 to 5.65	0.80
Male (vs. female)	5.36	−1.96 to 12.68	0.14
Age (per year)	−0.03	−0.26 to 0.19	0.75
ISS score	0.28	0.05 to 0.51	0.01
Charlson Comorbidity Index	4.57	−0.05 to 9.20	0.05

**Table 7:** Multivariable linear regression analysis of factors associated with mechanical ventilation duration.

Variable	Coefficient	95% CI	*p*-Value
Pre-QIP group (vs. post-QIP)	−3.34	−11.51 to 4.84	0.41
Male (vs. female)	2.70	−6.58 to 11.99	0.55
Age (per year)	−0.15	−0.44 to 0.13	0.28
ISS score	0.31	0.02 to 0.61	0.03
Charlson Comorbidity Index	6.97	0.08 to 1.55	0.03

## Data Availability

Data available from the corresponding author.
